# Symptoms and impacts of ambulatory nonsense mutation Duchenne muscular dystrophy: a qualitative study and the development of a patient-centred conceptual model

**DOI:** 10.1186/s41687-021-00341-x

**Published:** 2021-08-21

**Authors:** Kate Williams, Ian Davidson, Mark Rance, Axel Boehnke, Katharina Buesch, Sarah Acaster

**Affiliations:** 1 Acaster Lloyd Consulting Ltd, 16 Upper Woburn Place, London, WC1H 0BS UK; 2 PTC Therapeutics Ltd, Building 2, Ground Floor, Guildford Business Park, Guildford, UK; 3 PTC Therapeutics Germany GmbH, Am Flughafen 1, 60549 Frankfurt am Main, Germany; 4 PTC Therapeutics Switzerland GmbH, Tower 2, Turmstrasse 28, CH-6312 Steinhausen/Zug, Switzerland

**Keywords:** Duchenne muscular dystrophy, Nonsense mutation, Qualitative, Conceptual model, Symptoms, Ataluren

## Abstract

**Background:**

Duchenne muscular dystrophy is a rare genetic neuromuscular disorder, which can result in early death due to disease progression. Ataluren is indicated for the treatment of nonsense mutation Duchenne muscular dystrophy, in ambulatory individuals aged two years and older. This study explored the symptoms and impacts of nonsense mutation Duchenne muscular dystrophy and experience with ataluren.

**Methods:**

Qualitative interviews were conducted with caregivers in the UK. Interviews were conducted by telephone, were recorded and transcribed. Data were analysed using thematic analysis and saturation was recorded.

**Results:**

Ten interviews were conducted with parents of individuals aged 4–19 years. Key symptoms included muscle weakness and muscle breakdown, which were associated with limitations in physical function and pain. These impacted individuals’ daily activities, social activities and emotional wellbeing. These concepts and relationships were illustrated in a conceptual model, along with positive and negative moderating factors. Experience with ataluren and changes since initiation with treatment were discussed.

**Conclusion:**

Individuals with nonsense mutation Duchenne muscular dystrophy experience a range of interrelated symptoms and functional issues which impact their broader health-related quality of life. Treatments which address this high unmet need have the potential to improve the health-related quality of life of these individuals.

**Supplementary Information:**

The online version contains supplementary material available at 10.1186/s41687-021-00341-x.

## Introduction

Duchenne muscular dystrophy (DMD) is a progressive, X-linked neuromuscular disorder caused by mutations in the dystrophin-encoding DMD gene [1]. It is characterised by progressive muscle degeneration, resulting in delayed motor milestones, loss of ambulation, and potentially fatal cardiac and respiratory complications [2]. Median life expectancy with ventilatory support is reported to range from 21 to 39.6 years, with a pooled median of 29.9 years [3]. In approximately 10–15% of cases, DMD is caused by a nonsense mutation in the DMD gene (nmDMD) [4].

Treatments for DMD aim to control symptoms and improve health-related quality of life (HRQoL). Corticosteroid therapy has been shown to improve muscle function for up to 12 months, and muscle strength for up to two years in individuals with DMD [5]. Ataluren 40 mg/kg/day is the only licensed treatment for nmDMD and is indicated for the treatment of nmDMD in ambulatory individuals aged ≥2 years in member states of the European Union [6] and the UK. It enables the protein-making apparatus in cells to move past the defect, allowing the cells to produce a functional dystrophin protein, and has been shown to delay loss of ambulation and age at worsening performance in timed function tests [7]. Research is ongoing into other potential treatments for DMD, but these are not yet licenced in Europe [8]. In order to develop and evaluate effective treatments, it is important to understand the extent of the unmet need in this population.

Very few qualitative studies have been conducted among individuals with DMD or their caregivers, the majority of which do not focus on the symptoms and impacts of DMD. A recent qualitative study in individuals with DMD identified a range of HRQoL themes including physical aspects, social relationships, daily activities, autonomy, identity, and feelings and emotions [9]. Others have examined one specific symptom, such as anxiety [10] and pain [11], reporting that each of these were prevalent in individuals with DMD. To-date, no qualitative studies have examined the overall experience of individuals with DMD with respect to symptoms and HRQoL impacts, and none have focused specifically on those who are still ambulatory, or have explored the impact of ataluren. The primary aim of this study was to explore the symptoms, impacts and challenges experienced by ambulatory individuals with nmDMD, and the impact of ataluren. A second aim was to develop a conceptual model outlining the relationships between these symptoms, impacts and challenges.

## Materials and methods

### Design and participants

Qualitative interviews were conducted with caregivers of ambulatory individuals with nmDMD treated with ataluren in the United Kingdom. The aim of the interviews was to explore the symptoms and impacts of nmDMD before treatment with ataluren, as well as experience with ataluren. The interviews were conducted with caregivers of individuals with nmDMD, as individuals treated with ataluren were either too young or considered by their caregiver to be insufficiently capable to participate in an interview.

### Study materials

A semi-structured interview guide (Supplementary file 1) was developed based on the published literature on the HRQoL of individuals with DMD and through consultation with clinical experts in the UK and Germany and two patient advocacy groups (PAGs) in the UK (Muscular Dystrophy UK and Action Duchenne). The clinical experts included two medically/surgically trained clinicians, with a combined clinical experience of more than 10 years, and medical affairs/clinical development experience of more than 15 years, with a focus in muscular dystrophies. The PAG input included feedback from a caregiver of an individual with DMD. The interview guide comprised mainly of open-ended questions on the individual with nmDMD’s diagnosis and symptoms, as well as the impacts on their daily life, HRQoL, and the impacts on their caregiver (not included in this paper). These questions were asked in relation to their experience both before and since their son started taking ataluren.

A background questionnaire was developed to collect socio-demographic information and information on the individual with nmDMD’s diagnosis and treatments (Supplementary file 2). This included questions about motor function, which allowed individuals to be characterised into one of three ambulatory health states according to the natural history model developed by the University of Leicester (early ambulatory: can rise from supine, stand and walk 10 m; late ambulatory: can stand and walk 10 m; transfers: can stand) [12]. Throughout this paper we use the term ambulatory to refer to these three health states.

Caregivers also completed the DMD-QoL v2 Proxy, a newly developed questionnaire designed to assess the quality of life of individuals with DMD from the perspective of caregivers [13]. As the final version of the questionnaire was not yet available at the point the study was conducted, a draft version was used. This version had 27 items, each asking about an aspect of quality of life in the last week (e.g. ‘In the last week he was in pain’). All items were scored on a 4-point scale from 3 = never to 0 = all of the time, and there was also a not applicable option. Mean total scores (range 0–81) and mean average scores (range 0–3) were calculated based on scoring instructions from the questionnaire developers, with higher scores indicating better quality of life.

### Ethics review and approval

This study was reviewed and approved by the WIRB-Copernicus Group Independent Review Board (tracking number: #20193514).

### Recruitment and interviews

Participants were recruited by PAGs using purposive sampling. The study was advertised via newsletters and social media. Interested individuals were encouraged to get in touch using the contact details provided. Participant inclusion criteria were (i) being the main caregiver (at least 50% of caring) of an individual with nmDMD treated with ataluren in the UK, (ii) aged 18 years or over, (iii) live in the UK, (iv) willing and able to provide informed consent. Participants were sent an information sheet, background questionnaire and DMD-QoL v2 Proxy to complete and return the questionnaires by email. Due to the limited pool of potentially eligible participants, all of those who were eligible were included and no additional sampling criteria were used.

Interviews were conducted by three interviewers (KW, NP and KG), all with postgraduate degrees in psychology (two master’s and one PhD) and more than 25 years combined qualitative research experience. None of the participants were known to the interviewers. All interviews were conducted by telephone between 10 February 2020 and 20 March 2020 (prior to the UK COVID-19 lockdown). Verbal informed consent was taken at the start of the interview and was recorded. Interviews followed the semi-structured interview guide and lasted around 90 min. Participants were informed that they could refuse to answer any questions they did not wish to answer and were given the opportunity to speak freely and honestly. If participants answered inconsistently, they were probed to clarify any ambiguities.

The interview recordings were transcribed, and the transcripts were de-identified using participant identification numbers and names were removed prior to analysis. These were stored on a secure server, separate from any participant names and contact details.

### Analyses

Data from the background questionnaire were summarised using descriptive statistics. Data from the interviews were analysed using thematic analysis in MAXQDA. As with the interviews, all researchers involved in the analysis had postgraduate degrees in psychology (two master’s and one PhD), with more than 30 years combined qualitative research experience. Two researchers (KW and NP), who also conducted the interviews, read all the transcripts and developed a coding framework based on the topics covered in the interview guide. They then independently coded the same transcript (C101) and discussed discrepancies. A third researcher reviewed and provided input (SA). The coding framework was revised following this discussion and the remaining transcripts were coded by KW (*N* = 1) and NP (*N* = 8). To enhance the trustworthiness of the research, after each transcript was coded, the two researchers discussed and additional amendments were made to the coding framework as needed. When codes were added or changed, the previously coded transcripts were reviewed, and the new codes were applied as needed. KW conducted a final quality check of all transcripts to ensure the completeness and accuracy of the coding.

The codes were then grouped into themes to describe the experience of living with ambulatory nmDMD before and after treatment with ataluren. A conceptual model was developed to illustrate the relationship between these themes. The symptoms and impacts were described in boxes and arrows were used to indicate the direction of the relationships between these symptoms and impacts. These relationships were based solely on the qualitative data.

Best practice in qualitative research is to keep conducting interviews until data saturation is reached. Data saturation has been defined as the point at which no new insights are obtained, or no new themes are identified in the data [14]. A saturation matrix was used to monitor the frequency of reported concepts across the interviews, where the concepts were listed in rows and the columns were the interviews in order of completion [15].

## Results

### Sample characteristics

Ten caregivers took part in the interviews, all of whom were parents to an ambulatory individual with nmDMD. The caregiver characteristics are shown in Table 1 and the characteristics of the individuals with nmDMD who they care for are shown in Table 2.

**Table 1:** Caregiver characteristics (*N* = 10)

Characteristic	Mean (SD)	Range
**Age (years)**	**44 (5.4)**	**26–52**
**Relationship to individual with nmDMD**		**N**
Father		5
Mother		5
**Ethnic background**		
White		10
**Education**		
O level/GSCE or equivalent		3
A Level or Highers		1
Higher education below degree level		0
University degree or higher		6
**Employment**		
Employed full-time		6
Employed part-time		2
Full-time homemaker/caregiver		2

*SD* standard deviation; *nmDMD* nonsense mutation Duchenne Muscular Dystrophy

**Table 2:** Characteristics of individuals with nmDMD (*N* = 10)

Characteristic	Mean (SD)	Range
**Age (years)**		
Current	11.5 (5.0)	4–19
At diagnosis with nmDMD	3.3 (1.0)	1.8–4.8
**Length of time on ataluren (years)**	4.4 (3.5)	0.3–11
**Current health state**		**N**
Early ambulatory (age range 4–19 years)		6
Late ambulatory (age range 11–15 years)		3
Intermediate (aged 18 years)		1

*SD* standard deviation

Three participants had missing data on the DMD-QoL v2 Proxy (range of missing items per participant = 0–4). The mean total summed score for those with complete data (*N* = 7) was 59.9 (standard deviation (SD) = 2.1, range 46–69). The mean average score for completed items for all participants (*N* = 10) was 2.1 (SD = 0.3, range 1.7–2.6).

The saturation matrices indicated that saturation had been reached, with 100% (26/26) symptoms and impacts being reported in the first 50% of the interviews (Table 3).

**Table 3:** Data saturation matrix for symptoms and impacts

	Participant number^a^									
	C102	C101	C103	C104	C105	C106	C108	C107	C109	C110
**SYMPTOMS**										
**Muscle-related**										
Muscle weakness	**S**		S	S	S	S	S			S
Muscle breakdown	**S**	S			S	S	S			S
**Physical function**										
Difficulty walking distances	**S**	S	S		S	S	S	S	S	S
Slower walking	**S**	S		S	S				S	S
Difficulty with stairs		P		**S**	S		P	S		S
Difficulty getting up off the floor	**S**	S	S	S	S	S				S
Poor balance	**S**				S	P				
Difficulty lifting/carrying	P	P	P	**S**	S	S	S	P		S
Difficulty writing/drawing		**S**		S	S	S	S		P	S
Difficulty with fine motor skills	P	P		**S**	S	S	S			P
**Fatigue/tiredness**	**S**	S			S	S	S	S	S	S
**Cognitive-behavioural**										
Learning/memory difficulties	**S**	S	P	S	S	S	P	P	P	
Difficulty focusing/concentrating	**S**	S			S	P	P		S	P
Communication/language difficulties			P	**S**	S	S				
Aggressive behaviour					**S**		S	S		S
Autistic/ADHD spectrum behaviours		**S**	S	S		S	S		S	S
**Pain/discomfort**										
Lower limb pain/discomfort	**S**	S			S	P	P	P		
Upper body pain/discomfort		**S**			P					
**IMPACTS**										
**Daily activities**										
School/nursery	**S**	S	S	S	S	S	S	S	S	S
Self-care	**S**	S		S	S	S	S	S	S	S
**Social activities**										
Difficulty keeping up with others		**S**		S	S	S	S	S	S	S
Missing out/avoiding social situations	**S**	S			S	P	S	S		S
**Emotional wellbeing**										
Upset/sadness		**S**		S	S	S	S	S	S	
Frustration	**S**	S			S	S		S		S
Anxiety	**S**						P	S	S	S
Confidence/self-esteem	P			**S**	S		P	S		

^a^Participant numbers are presented in the order the interviews were conducted. S = spontaneously reported impacts, P = probed impacts. Impacts were considered spontaneously reported unless explicitly probed. Bold letters indicate the first spontaneous report

### Overview of symptoms and impacts before treatment with ataluren

**Fig. 1:**
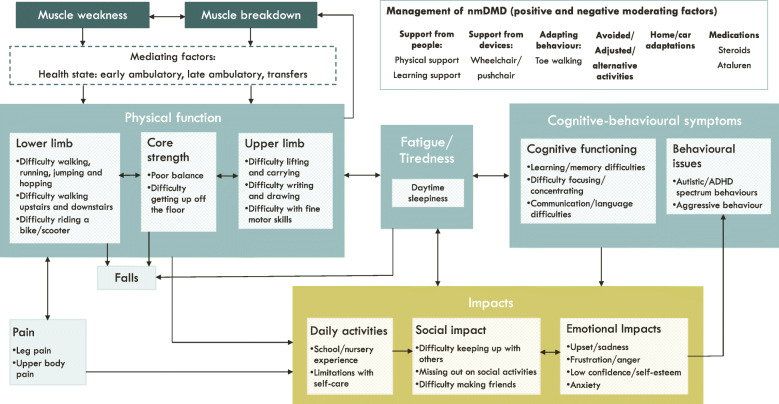
Conceptual model illustrating the relationships between symptoms and impacts before treatment with ataluren

The symptoms and impacts and relationships between them are shown in a conceptual model in Fig. 1. This shows the symptoms and impacts reported by caregivers when asked about the period *before* their son started taking ataluren. Muscle weakness and muscle breakdown were at the core of the symptoms and issues reported by caregivers, resulting in impaired physical function. The health state (level of ambulation) of the individual with nmDMD mediated the relationship between muscle weakness and breakdown and physical function, with those in the later health states having more impairments in physical function. These impairments in physical function often resulted in fatigue/tiredness, pain and falls, which in turn had a further negative impact on physical function. Caregivers also reported that fatigue was a core symptom of nmDMD and that this impacted their son’s physical function and cognition and behaviour. All of the reported symptoms had an impact on three key areas of life, including daily activities, social activities, and emotional wellbeing. These emotional impacts were in turn linked to some of the behavioural issues. These are described in further detail in the sections below.

### Symptoms and function before treatment with ataluren

#### Muscle weakness and breakdown

Most caregivers spontaneously reported muscle weakness as a core symptom of nmDMD.

*“Definitely the muscle weakness side of things, not being able to walk for long periods of time, not being able to run and play, suffering with fatigue every day and just physically just not being able to do what other kids of his age could do”* – C108.

They described how their sons lacked core, lower and upper limb strength and that this impacted every aspect of their physical function, including their ability to get up off the floor, walk and move around, and use their arms. Some caregivers described how their sons had very dark urine on days when they had been particularly physically active, which they interpreted as a sign that their muscles were becoming damaged and breaking down.

*“I took him to a party … he was so exhausted at the end of it … it was a really bad idea because then he went to have a wee after he came home, his wee was very dark which is a sign of muscle breakdown”* – C101.

This sometimes led them to limit their sons’ activities to avoid their muscles becoming more damaged and further hindering their physical function.

#### Physical function

All caregivers discussed the impact of nmDMD on their son’s ability to walk. Although one individual had recently lost the ability to walk (transfers health state), their caregiver was still able reflect back on their walking prior to this happening. Nearly all caregivers described how their son was unable to walk very far or for very long as they would become very tired and need to rest. Some caregivers mentioned that they used a pushchair or a wheelchair, not necessarily because their son was unable to walk the distance, but to give them the opportunity to walk for some of the time and then rest.

*“We couldn’t go places where we had to walk a long way, because he just got so tired. We used to walk him to nursery, which is about I’d say less than quarter of a mile, and he used to have to sit down two or three times on a wall, just to get his breath back, rest his legs”* – C105.

Several caregivers reported that although their son could walk, they were much slower than other children their age, which made it difficult for them to keep up with their friends. Most reported that their son was unable to run, although some described that their child had a “Duchenne waddle”.

*“It’s known as a Duchenne waddle, where the arms are out and the back’s arched and they’re like a penguin, the way a penguin walks. Where they’re trying to keep their balance and walk as well. But you could see how much thought had to go into just walking for him to get from one room to the other. And when I say running, it weren’t running, it was more of a waddle”* – C105.

Stair climbing was a challenge, with several caregivers reporting that they needed to carry their child up and down stairs, particularly when they were tired.

*“He couldn’t climb stairs as well and that’s always been the case so we’ve always helped him upstairs”* – C106.

Most caregivers said that their sons had difficulty getting up off the floor unaided due to poor core strength. Some described how they used the Gowers’ sign (a manoeuvre where the individual has to use their hands and arms to “walk” up their own body from a squatting position due to lack of hip and thigh muscle strength).

*“If he was getting off the floor, he would be able to get off the floor but he would be doing the, like the Gowers’ movement, where he’d be doing it, using his hands on his legs and pulling himself up that way”* – C101.

A few caregivers mentioned that their son had poor balance which resulted in clumsiness and frequent falls. Others attributed the falls to their son being tired and finding it harder to walk.

*“It was hard for him to keep his balance, it was hard for him to steady himself”* – C102.

Most caregivers reported that their sons needed help with lifting and carrying heavy objects due to their muscle weakness.

*“If we gave him a bag of sugar, he wouldn’t have been able to pick it up, because that would have been just too much for him, he didn’t have the core strength in his upper body to lift it”* – C105.

Others described how their sons struggled with writing or drawing as they did not have the strength to press down hard enough with a pencil.

“*( …*) *He would struggle to hold a pencil and write. He’d write very lightly, if that makes sense? So, not able to sort of, so if he was drawing, you could barely see it, it would be so light because he wasn’t pressing down”* – C101.

Difficulties with fine motor skills were also reported, with several caregivers saying that their sons were unable to do up a button or use a knife and fork. However, some said that they would be able to feed themselves with a fork once the food had been cut up.

*“He couldn’t do up a button no, he couldn’t get himself dressed. He couldn’t put his shoes on, he couldn’t put a sock on. He struggled to put a hat on his head “*– C104.

#### Pain/discomfort

Physical function had an impact on pain, most commonly in the legs. Most caregivers reported that their sons complained of muscle aches, cramps and pain in their legs after walking or other exercise.

*“He would just complain that his legs were just hurting probably from the knee down, his calves basically, that they would be hurting if he had to do any significant amount of walking”* – C101.

Most caregivers attributed this to overexertion and their sons becoming fatigued and needing to rest, with one caregiver noting that the pain was a potential indicator of muscle damage. This leg pain had a subsequent impact on their physical function, as they became less able to move around once their legs started hurting. One caregiver also reported that their son had pain in their arms, although this was less common.

#### Fatigue

Fatigue was another commonly reported symptom and was a key feature of what caregivers described as a ‘bad day’ for their sons. Several caregivers said that their sons became very fatigued after a busy day at school, or when they had spent a lot of time walking or playing.

*“He would fatigue very quickly, so he wouldn’t, he wasn’t able to walk very far or climb or run, despite him being cautious but he would also fatigue very quickly”* – C106.

This physical fatigue frequently led to them complaining that their legs were aching, resulting in a need to stop activities to rest and be massaged regularly. Some said that their son became more prone to falls when they were fatigued, putting them at risk of injury. One caregiver described how days were often a cycle of activity and rest, as their son wanted to be active but would become fatigued easily. Some caregivers reported using a pushchair or wheelchair to allow their son to rest when they became fatigued. Two caregivers mentioned that their sons sometimes became so fatigued that they fell asleep after coming home from school or needed to nap during the day. As well as the physical fatigue, some caregivers described how their sons became upset or exhibited difficult behaviours when they were fatigued.

#### Cognitive-behavioural symptoms

Most caregivers reported that their sons had learning difficulties and struggled to remember things they had been taught.

*“He can remember things but it’s in his learning if he, he’s learning say a maths thing, he might quickly forget that, whereas things in everyday life and memories, he can remember things”* – C103.

Others reported difficulties with reading and spelling as well as with numbers. Often related to learning difficulties, several caregivers reported that their sons had trouble focusing and concentrating, often describing them as having a short attention span. This was most often discussed in terms of their ability to concentrate on schoolwork or homework, but one caregiver also reported that their son had a lack of focus when choosing a toy to play with.

*“He was getting a little bit grouchy about the fact that people kept on asking him to concentrate on something, on a piece of work, or even at home doing a bit of homework he would be struggling to focus on it”* – C105.

Some also described how the individual struggled with verbal communication and language.

*“Yeah, he used to do a lot of indicating with his hands, because as I say communication verbally wasn’t very good. And the indication was hands pointing to his legs, and we’d have to massage them”* – C105.

Some caregivers reported that their sons could sometimes become aggressive and lash out at others. This was generally described as being linked to frustration at not being able to do certain activities or struggling with schoolwork. Some caregivers described their sons’ behaviours as being on the autistic or ADHD spectrum, which meant that they struggled in certain social situations and could have emotional outbursts.

### Impacts of nmDMD before treatment with ataluren

#### Daily activities

The two main impacts of nmDMD on daily activities were on school (or nursery/college) and ability to self-care. All caregivers in this study had sons who attended school and this was perceived as an important component of what constituted a good day for them. Although school was generally reported as a positive experience for individuals with nmDMD, it was associated with a range of challenges. Due to their learning difficulties and difficulties with concentration, many individuals struggled with the actual schoolwork and required one-to-one classroom support.

*“He would struggle in school … rather than do an hour lesson he would do 20 minutes at a time, have a break and then go back to it. But, he had full one-to-one support at school so they could manage that because he would struggle with some behaviours. If he was tired, he could be quite, he would play up a little bit towards other students and maybe make comments”* – C108.

Several caregivers reported that their son’s behaviour at school could be difficult to manage and they could be quite disruptive in class. Some reported that their sons would sometimes become aggressive towards other children. This disruptive behaviour was often attributed to their son being frustrated or upset at not being able to focus or participate in certain activities. Several caregivers also reported that their sons found school very tiring and that this could also have a negative impact on their behaviour.

All caregivers reported that their sons needed significant help with self-care, although this varied considerably, partly due to age. Due to their son’s limitations with physical function, some caregivers needed to help their sons with all aspects of self-care.

*“We would have to get up in the morning to do medications and then it was to do physio and then to help him get washed and dressed and help him get out and get ready for school, in and out of bath, in and out of the car, things that as I say you don’t really realise that you’re doing, compared to what you would do with a non-Duchenne child”* – C102.

Others reported that their sons were able to do some tasks independently, for example, one individual was able to use the toilet himself as their house had been adapted to create an accessible bathroom. Another individual was able to largely dress himself, but needed help with shoes and socks. Some individuals could eat independently, but would need help with cutting up food.

#### Social activities

Three main social impacts were identified; difficulty keeping up with others, missing out on activities and making friends. Most caregivers reported that their son’s limitations in physical function impacted their ability to keep up with their peers.

*“I’ve seen it a couple of times, where they’d all rush out into the playground, and he’d just be sitting there up against the wall in the corner, waiting for them to all rush past him, and then he’d sort of waddle out as well”* – C105.

In addition, most caregivers reported that their sons had to miss out on certain activities completely as they were either physically unable to take part or they avoided letting them take part for fear they would injure themselves or become too fatigued. For example, several participants mentioned that they avoided bouncy castles as their son was unable to jump and so would become upset at not being able to join in.

*“He’s not meant to go on a bouncy castle … he would tend to miss out on certain things, so if there was a party after school for example on a Thursday then we would tend not to take him because he’d be so tired, he’d be struggling so much”* – C101.

Not being able to keep up with their peers and missing out on activities were most frequently reported as having the biggest impact on their son’s quality of life. As well as struggling to keep up physically, some caregivers reported that their sons found it difficult to make friends because of their behaviour, as they struggled to interact with their peers appropriately.

*“He doesn’t have as many friends because his physical disability is far more apparent, so a lot of his friends would go off to the astro pitch and play football, and he’s not left with much of a choice of what he can do. So, he needs to make more friends who aren’t into the physical activities”* – C107.

#### Emotional wellbeing

Most caregivers reported that their son’s emotional wellbeing was impacted by their nmDMD. The most commonly reported emotion was upset or sadness. This was most commonly attributed to not being able to join in with peers or take part in certain activities.

*“We don’t go to sports day anymore, we get the day off from school because it’s, that’s been for three years now actually we’ve not been going to sports day or he went to one, no they made us go to one and then I just said, they got him really involved, he did a few sports but they got him involved with helping to manage it and do scoreboards and stuff like that but he was still just, “This is just a big memory of what I can’t do and I don’t want to be here” and that night he was just in pieces because he was like, “I hate it, I absolutely hate it” basically so I said, “He’s never coming again” which they accepted basically”* – C104.

Several caregivers described how their son would often become upset when they were particularly tired. Another commonly reported emotion was frustration at not being able to do something. This was sometimes described in relation to schoolwork, where their son may struggle with concentration and then become frustrated or angry and then their behaviour would become more difficult to manage. Other times it was in relation to their limitations in physical function, for example, not being able to get up off the floor to go and get a toy. This frustration was sometimes linked to emotional outbursts, where they would become angry and upset.

*“I think he really showed his frustration with anger and I would say that myself and his older brother really took the heat of that. And I think it was pure frustration that he was unable to do as much physically as his brother and his friends … For example, if him and his brother played football, he wouldn’t be able to keep up and he would get cross and either hit [brother’s name] or he’d just not want to play anymore”* – C107.

A smaller number of caregivers reported that their son could be anxious and this was generally linked to their behaviour issues.

*“He would have anxiety but he wouldn’t show it. It was like an internal battle with himself. I think because of the autistic spectrum, he wasn’t good in determining that emotional side of things and knowing what those feelings were. So, he would have anxiety but he wouldn’t quite understand what that was and we could be unaware of it for many days until he would have an outburst. A good example is if there was something like a birthday of his or Christmas coming up, there would always be some sort of emotional incident, like he’d have a really bad day at school and abuse a teacher or a child verbally. Excitement would cause that level of anxiety”* – C108.

Others reported that their sons had low confidence and self-esteem because they were unable to do things that other people their age were able to do.

*“It would impact his confidence and self-esteem quite a lot. He would criticise himself, “Oh I’m not good enough for that, I can’t do that” or, “Why can’t I do this?” and, “It’s not fair”, that type of stuff and, “Why me?” Yeah, it definitely impacted his confidence and his self-esteem”* – C108.

### Management of nmDMD

Caregivers described a range of moderating factors which could have a positive and/or negative impact on the symptoms and impacts of nmDMD. Several reported that their child received some kind of professional support which helped them manage their nmDMD. This included learning support at school, as well as healthcare support. Some described how their son used equipment or devices to help them cope with limitations in physical function, most commonly the use of a wheelchair or pushchair. While this was helpful in terms of preventing fatigue or pain from overexertion, there were also occasions when it was reported to have a negative impact, for example, missing out on an activity that was not wheelchair accessible.

**Positive impact:***“He could still walk unaided, just not long distances, I mean he couldn’t go on like a 15/20 minute walk, maybe five/10 minutes and then look to get into the wheelchair but more so to preserve function and more so that he wouldn’t be overexerted and have that muscle breakdown”* – C102

**Negative impact:***“If they had a school trip, the school trip had to be adapted so that he would be able to participate in the school trip. If they were going to a soft play area or going to a beach, his wheelchair wouldn’t have went through the sand”* – C102

Some caregivers mentioned that they had needed to move to a more practical house or make home renovations to make it easier to manage their son’s nmDMD.

*“He has got a room now which is ensuite with a wet room, he’s got a toilet that he can keep his independence in the future with … we have ramps at the house … we have a lift”* – C107

Others described how their sons had adapted their behaviours to accommodate their limitations in physical function, for example, by coming down the stairs on their bottom.

“If it was a couple of steps, a few little steps, then he would crawl up them and then come down on his bottom” – C101

### Experience with ataluren

Several caregivers reported that they had noticed positive changes in their son’s symptoms or level of function since they had started taking ataluren. This included improved muscle strength, improvements in the length of time they could walk, reduced fatigue/increased energy levels and improvements in concentration.

*“Yesterday again, for example, he got out of his all-terrain hopper and he walked for I would say a good 20 minutes or more yesterday. Without Translarna, I don’t think he would be able to do that”* – C107

*“His ability to climb and descend stairs improved”* – C110

*“He’s definitely got more energy so that’s less of a concern for me and my husband really as parents”* – C101

*“We didn’t expect to see him, things like his concentration improving so quickly and actually starting to learn, finding learning an easier process because he’s able to concentrate more and things like that”* – C101

Improvements in impacts were also reported, with some caregivers reporting that their social interactions improved and others noticing an improvement in their son’s emotional wellbeing.

*“We’re able to take [name of individual with DMD] to more places and do more things and have friends, school friends over etc. so from that perspective it’s meant that we could expand our social interaction”* – C110

*“We don’t get the outbursts like we did for the, so I’d say the frustration and the anger isn’t as acute*” – C107

Other caregivers said that they had not noticed any changes since their son had started taking ataluren. Although one caregiver viewed this negatively, others perceived the stability of symptoms to be a positive.

*“It’s just good to see that [he] can be stable. Obviously we know that things will change at some point but it’s a much slower decline so it gives you just more time to play with really and it’s just positive all round”* – C108

*“It’s getting it across that, just because he’s not able to run a marathon now, doesn’t mean it’s not working. Maintaining the function is just as important”* – C102

*“I don’t think [ataluren] has any effect on him, I don’t think it works, I don’t think it has any impact*” – C103

Some caregivers reported that their son’s symptoms and physical function had declined since starting ataluren, but this was generally attributed to the natural course of nmDMD. One caregiver said that even though their son’s nmDMD had progressed, they still thought that ataluren had delayed the progression.

*“The drug has slowed the progression of the condition enormously but because of the nature of the condition, he’s now worse than he was four years ago or three years ago or two years ago”* – C106

*“He doesn’t get upstairs now, I have to carry him up the stairs”* – C104.

## Discussion

This is the first qualitative study to explore the symptoms, impacts and challenges experienced by ambulatory individuals with nmDMD and to illustrate the relationship between these concepts in a patient-centred conceptual model. These findings highlight a significant unmet need among individuals with nmDMD. Although some of these concepts have previously been reported in the literature [14–17], new concepts were identified in this study, highlighting issues that are potentially specific to ambulatory individuals with nmDMD, which have not previously been explored. In addition, the development of a conceptual model extends these findings by highlighting the relationships between these concepts. This study also provided novel insights on the experience of treatment with ataluren. While improvements cannot be attributed to ataluren based on this study, the reports are consistent with some individuals still being ambulatory in their late teens, when the typical progression of DMD results in loss of ambulation at around 12 years [16].

The patient-centred conceptual model was directly based on the important concepts reported by caregivers in this study. Given the relationship between the concepts highlighted in the conceptual model, these findings highlight the potential impact of a treatment to extend beyond core symptoms to the broader HRQoL of individuals with nmDMD. For example, an improvement in muscle strength may improve physical function, which has the potential to lead to an improvement in pain, falls, fatigue/tiredness, daily activities, social activities and emotional wellbeing. As well as highlighting the symptoms and impacts of nmDMD and the potential impacts of treatment, the conceptual model can provide a useful tool for informing a patient-centred measurement strategy for clinical trials of the evaluation of new treatments. As there are currently a lack of patient-reported outcomes (PROs) that have been validated for use in DMD [17], this conceptual model can be used as the basis for developing or validating patient-centred outcome measures for the specific ambulatory population included. For example, the conceptual model highlights that physical function, fatigue/tiredness and cognitive-behavioural symptoms are key concepts that would be important to capture in clinical trials to evaluate treatments for nmDMD.

While this study provides novel insights, it also had a number of limitations that need to be acknowledged. All participants in this study were caregivers of individuals with nmDMD who were currently taking ataluren. While this was necessary for exploring the impact of ataluren, ideally the study would also have included those not on ataluren, as those eligible for treatment may have different characteristics to those who are not eligible. In addition, participants had to rely on retrospective recall when asked about their son’s experience prior to taking ataluren, which is not as reliable as including caregivers of individuals not yet on treatment. Although data saturation was reached, the sample size is limited so it is possible that the findings may vary in different populations. Finally, the interviews were conducted with caregivers of individuals with nmDMD rather than the individuals themselves and they may not be fully able to describe the full impact of nmDMD.

Limitations aside, this is the first qualitative study to provide an in-depth exploration of the symptoms and impacts of ambulatory nmDMD. Future research could focus on expanding the qualitative study to include caregivers or individuals with nmDMD who are non-ambulatory, to better understand the symptoms and impacts of this population and the potential benefits of delaying disease progression. The results may also be used to inform the development of prospective surveys to explore the impact of nmDMD in a larger sample. Such studies could also be used to quantitatively evaluate the impact of treatment with ataluren using validated PROs.

## Conclusions

Ambulatory individuals with nmDMD experience a range of interrelated symptoms and functional issues which negatively impact their HRQoL. Treatments which address this high unmet need by improving symptoms, function, or delaying progression have the potential to improve HRQoL in these individuals.

## Supplementary Information

**Additional file 1:.** Interview guide

**Additional file 2:.** Background questionnaire

## Data Availability

Available from corresponding author upon request.

## Abbreviations

DMD: Duchenne muscular dystrophy
nmDMD: Nonsense mutation duchenne muscular dystrophy
HRQoL: Health-related quality of life
PAG: Patient advocacy group
PRO: Patient-reported outcome
SD: Standard deviation
